# In Vitro and In Vivo Effects of Ulvan Polysaccharides from *Ulva rigida*

**DOI:** 10.3390/ph16050660

**Published:** 2023-04-28

**Authors:** Jorge García-Márquez, Bruna Rodrigues Moreira, Piedad Valverde-Guillén, Sofía Latorre-Redoli, Candela T. Caneda-Santiago, Gabriel Acién, Eduardo Martínez-Manzanares, Manuel Marí-Beffa, Roberto T. Abdala-Díaz

**Affiliations:** 1 Department of Microbiology, Faculty of Science, Andalusian Institute of Blue Biotechnology and Development (IBYDA), Malaga University, Campus Universitario de Teatinos s/n, 29071 Malaga, Spain; 2 Phycology Laboratory, Department of Botany, Biological Sciences Center, Federal University of Santa Catarina, Florianópolis 88040-900, SC, Brazil; 3 Department of Cell Biology, Genetics and Physiology, Faculty of Science, Andalusian Institute of Blue Biotechnology and Development (IBYDA), Malaga University, Campus Universitario de Teatinos s/n, 29071 Malaga, Spain; 4 Department of Chemical Engineering, Almería University, 04120 Almería, Spain; facien@ual.es; 5 Instituto de Investigación Biomédica de Málaga-IBIMA, Hospital Universitario Virgen de la Victoria, Universidad de Málaga, 29071 Málaga, Spain; 6 Networking Biomedical Research Centre in Bioengineering, Biomaterials and Nanomedicine (CIBER-BBN), Málaga Biomedical Research Institute and Nanomedicine Platform (IBIMA BIONAND Platform), 29071 Málaga, Spain; 7 Department of Ecology and Geology, Faculty of Science, Andalusian Institute of Blue Biotechnology and Development (IBYDA), Malaga University, Campus Universitario de Teatinos s/n, 29071 Malaga, Spain

**Keywords:** cytotoxic activity, human cancer cell lines, polysaccharides, *Ulva rigida*, ulvan, zebrafish embryo toxicity test

## Abstract

One of the main bioactive compounds of interest from the *Ulva* species is the sulfated polysaccharide ulvan, which has recently attracted attention for its anticancer properties. This study investigated the cytotoxic activity of ulvan polysaccharides obtained from *Ulva rigida* in the following scenarios: (i) in vitro against healthy and carcinogenic cell lines (1064sk (human fibroblasts), HACAT (immortalized human keratinocytes), U-937 (a human leukemia cell line), G-361 (a human malignant melanoma), and HCT-116 (a colon cancer cell line)) and (ii) in vivo against zebrafish embryos. Ulvan exhibited cytotoxic effects on the three human cancer cell lines tested. However, only HCT-116 demonstrated sufficient sensitivity to this ulvan to make it relevant as a potential anticancer treatment, presenting an LC_50_ of 0.1 mg mL^−1^. The in vivo assay on the zebrafish embryos showed a linear relationship between the polysaccharide concentration and growth retardation at 7.8 hpf mL mg^−1^, with an LC_50_ of about 5.2 mg mL^−1^ at 48 hpf. At concentrations near the LC_50_, toxic effects, such as pericardial edema or chorion lysis, could be found in the experimental larvae. Our in vitro study supports the potential use of polysaccharides extracted from *U. rigida* as candidates for treating human colon cancer. However, the in vivo assay on zebrafish indicated that the potential use of ulvan as a promising, safe compound should be limited to specific concentrations below 0.001 mg mL^−1^ since it revealed side effects on the embryonic growth rate and osmolar balance.

## 1. Introduction

According to the World Health Organization, cancer was the second leading cause of death globally in 2020 [1]. Breast cancer is the most prevalent cancer worldwide, whereas colorectal cancer, leukemia, and skin melanoma rank third, thirteenth, and seventeenth amongst the main forms of cancer, according to the Global Cancer Observatory (GCO) of the International Agency for Research on Cancer (IARC) (gco.iarc.fr). The frequency of this disease is expected to rise to more than 30 million people by 2040, a 56% increase from 2020 (gco.iarc.fr). Effective therapy strategies remain limited even though significant breakthroughs have been made in understanding the different pathways that drive the emergence of cancer. These strategies entail a combination of therapies, such as chemotherapies, which use various chemicals to target malignant and healthy cells [2,3]. Despite promising improvements in targeted therapeutics [4,5], their efficiency is restricted in some situations due to drug resistance [6,7].

Natural product research has emerged as a serious alternative for finding new bioactive compounds, with seaweed being one of the most promising sources of therapeutic candidates due to the vast richness of the marine environment [8]. Seaweeds, which live in harsh environmental and ecological circumstances, have been used for various purposes, including feed, food, and biotechnological applications [9]. This is due to their bioactive and nutritious compounds, which make them a valuable resource [10,11]. Green macroalgae species of *Ulva* are distributed around the world and are capable of occupying diverse habitats because of their tolerance to determinant factors, such as light, temperature, and salinity [12]. They present high growth rates and productivity under very variable conditions, having highly exploitable biochemical profiles [13], including bioactive metabolites that are of interest with regard to many economic applications, such as food, feed, fertilizers, and biomedicine [14].

One of the main bioactive compounds present in *Ulva* species is the sulfated polysaccharide ulvan. The ulvan complex structure varies according to the algae species, the growing location and conditions, and the extraction procedures [15]. Ulvans constitute between 8 and 29 % of the dry weight depending on the *Ulva* species and growing conditions [16]. These complex sulfated polysaccharides are interesting in terms of biomedical applications due to their antioxidant, antitumor, anticoagulant, antiviral, anti-inflammatory, and immune-modulator properties [17,18,19,20]. Recent attention has been given to the anticancer properties that ulvans possess because ulvans obtained from different *Ulva* species have demonstrated significant cytotoxic activity against hepatocellular carcinoma (HepG2), human breast cancer (MCF7), human colon carcinoma (HCT-116), and cervical cancer (HeLa) cells [21,22,23,24]. Their anticancer activity seems to operate via different pathways, including promoting cancer cell apoptosis, reducing cancer cell proliferation, and stimulating the innate immune response [14]. Furthermore, the pathways affected depend on the source and/or structure of the ulvans [15]. Therefore, specific research should be conducted on each *Ulva* species and cancer cell line. 

Polysaccharide extracts have also been tested on living organisms, for example, on zebrafish embryos, in the so-called zebrafish embryo toxicity test (ZFET) [25]. Zebrafish have become an alternative model to rodent toxicity in in vivo assays [26,27,28]. This model provides important features, namely, rapid external embryonic development, a small size, optical transparency, a large number of offspring, and genetic similarities to humans [29,30]. Furthermore, zebrafish have been reported to have functional homologs for more than 90 of the 450 human genetic dysplasias [31]. 

Several interesting tests that use these embryos have also been proposed, such as rapid, high-throughput, cost-effective drug and chemical screening tests [29,32,33,34,35]. Zebrafish embryos have already been used to test for beneficial fungicidal [36], antioxidant [37,38,39,40,41], anti-inflammatory [40,42,43,44,45,46], immunomodulatory [47,48,49,50], genoprotective [51], hepatoprotective [52], disease-resistant [49], and antitumor activities [53]. Conversely, these tests have been employed to show the detrimental toxic effects of drugs and chemicals [25,54,55,56,57] leading to limitations in their potential clinical or veterinary use. Among these studies, several works specifically analyzed the effects of algal polysaccharides on zebrafish embryos [39,43,44,58].

In this study, we obtained ulvan polysaccharides from the green macroalga *Ulva rigida*. The in vitro antitumor activity of the ulvans was evaluated with MTT assays using healthy cell lines (1064sk (human fibroblasts) and HACAT (immortalized human keratinocytes)) and carcinogenic cell lines (U-937 (a human leukemia cell line), G-361 (a human malignant melanoma), and HCT-116 (a colon cancer cell line)). In addition, the cytotoxic activity of the ulvans was evaluated using a zebrafish embryo toxicity test (ZFET) [25].

## 2. Results

### 2.1. Ulvan Composition and Structure

#### 2.1.1. Fourier-Transform Infrared Spectroscopy (FTIR)

**Figure 1:**
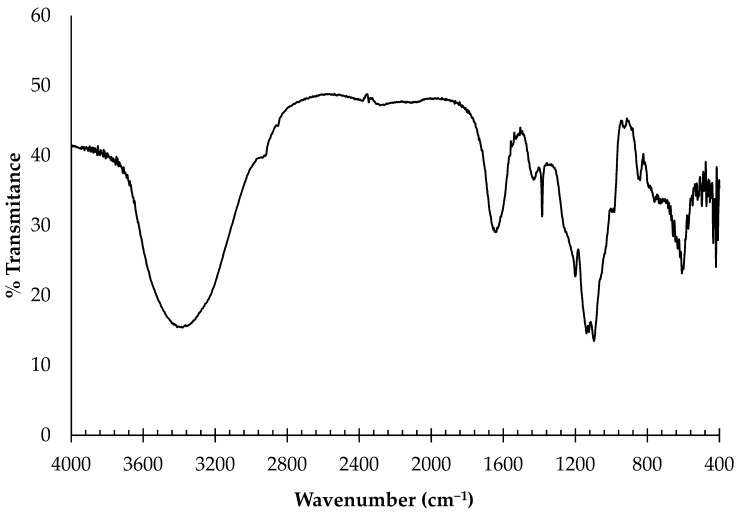
FTIR spectroscopy of ulvans from *U. rigida*.

FTIR spectroscopy of the ulvans from *U. rigida* showed the presence of several functional groups (Figure 1). The strong broad absorption band centered at about 3402 cm^−1^ corresponds to the hydroxyl group (OH) stretching vibration. The weak absorption at 2938 cm^−1^ was due to the stretching vibration of C–H. Two other bands were observed between 1650 and 1430 cm^−1^, characteristic of the carboxylate groups of uronic acids in the ulvan. The strong absorption at 1640 cm^−1^ was ascribed to the asymmetric stretching mode of the COO– group, and weaker absorption around 1438 cm^−1^ arose from the symmetric COO– stretching mode. The most important absorptions were those revealed at approximately 1260 cm^−1^ and 1056 cm^−1^, considered the fingerprint region for ulvan [59]. A moderate absorption at the 1200 cm^−1^ wavelength is characteristic of the stretching vibration of the polysaccharide’s sulfate ester (S=O), referring to the C–O stretching of the two principal sugars, namely, rhamnose and uronic acid. The absorption peaks at about 850 cm^−1^ correspond to the C–O–S bending vibration of sulfate in the axial position.

#### 2.1.2. Gas Chromatography–Mass Spectrometry (GC–MS)

**Figure 2:**
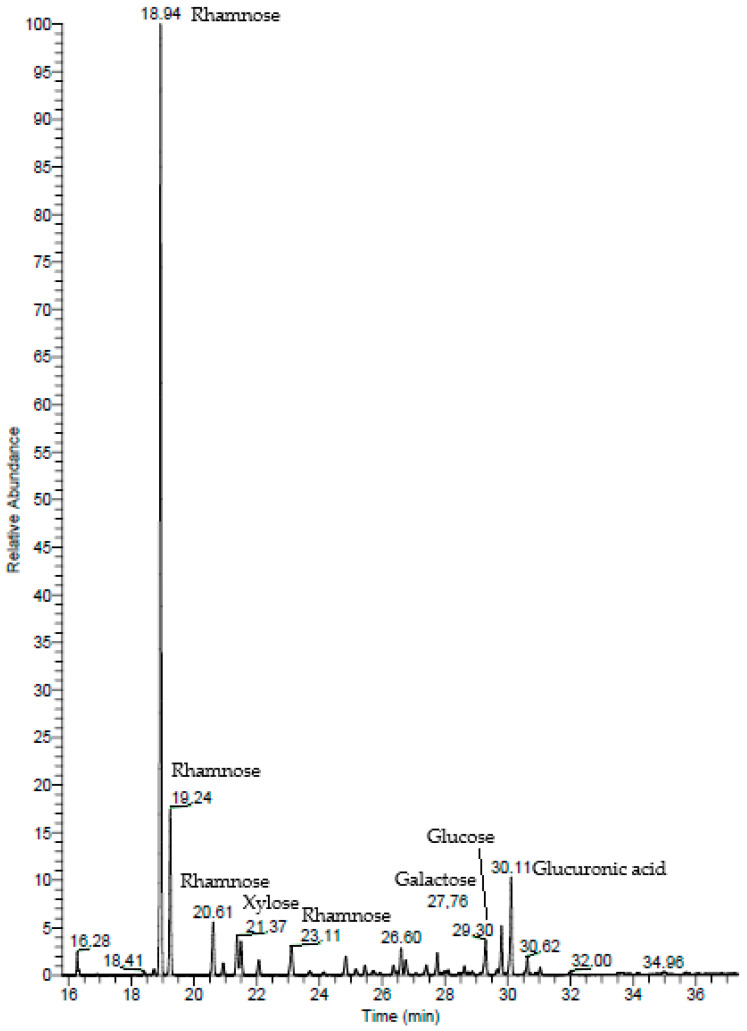
Gas chromatography–mass spectrometry (GC–MS) of ulvans from *U. rigida*.

In the GC–MS spectrum of the ulvans from *U. rigida*, the highest peak corresponds to rhamnose, followed by glucuronic acid and xylose (Figure 2). The rhamnose, glucuronic acid, and xylose percentages were 80.60%, 9.14%, and 4.01%, respectively (Table 1). Other monosaccharides, namely, glucose and galactose, were also detected (3.78% and 2.48%, respectively).

### 2.2. Cytotoxic Activity of Ulvan Polysaccharides

**Figure 3:**
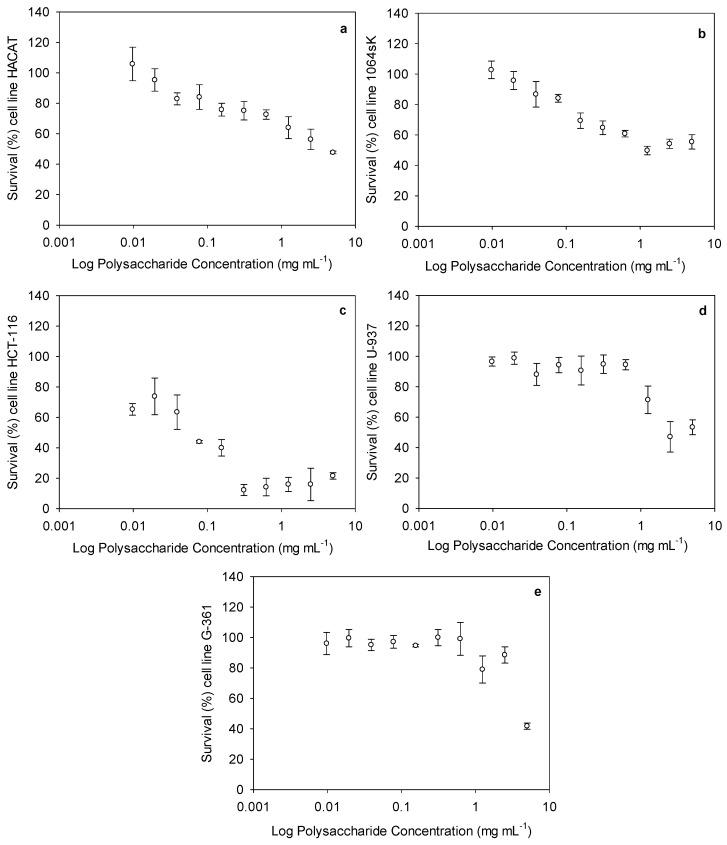
Cytotoxic activity of ulvan polysaccharides, expressed as survival (%) of each cell line depending on the ulvan polysaccharide concentration (mg mL^−1^). Each figure represents a cell line: (**a**) immortalized human keratinocytes (HACAT), (**b**) human fibroblasts (1064SK), (**c**) human colorectal carcinoma cell line (HCT-116), (**d**) human myeloid leukemia (U-937), and (**e**) human malignant melanoma (G-361).

The cytotoxic activities of the ulvans at different concentrations (ranging from 0.009 to 5 mg mL^−1^) against the healthy and carcinogenic cell lines are presented in Figure 3. With regard to the cytotoxicity against the healthy cells, a lower cytotoxic effect was observed in the keratinocyte (HACAT) cells, presenting an IC_50_ value of 4.2 ± 0.5 mg mL^−1^, than in the fibroblast (1064sK) cells, which exhibited an IC_50_ of 1.2 ± 0.1 mg mL^−1^ (Figure 3a,b). For the carcinogenic cells, the IC_50_ values were estimated for the colon (HCT-116), leukemia (U-937), and melanoma (G-361) cells as being 0.1 ± 0.02, 2.4 ± 0.4, and 4.3 ± 1.2 mg mL^−1^, respectively (Figure 3c–e).

After obtaining the IC_50_ values, the selectivity index (SI) was also calculated (Table 2). The SI is the ratio obtained by dividing the IC_50_ value of the healthy cells by that of the cancer cells. The higher the SI, the more effective and safer a drug would theoretically be during in vivo treatment. The selectivity between the healthy and cancer cell lines varied for our compound. The highest selectivity indexes were estimated as 40.9 and 11.5, respectively, for the ulvans used against the HCT-116 cells compared to the HACAT and 1064sk cells.

### 2.3. Zebrafish Exposure to Increasing Concentrations of Ulvan Polysaccharides

**Figure 4:**
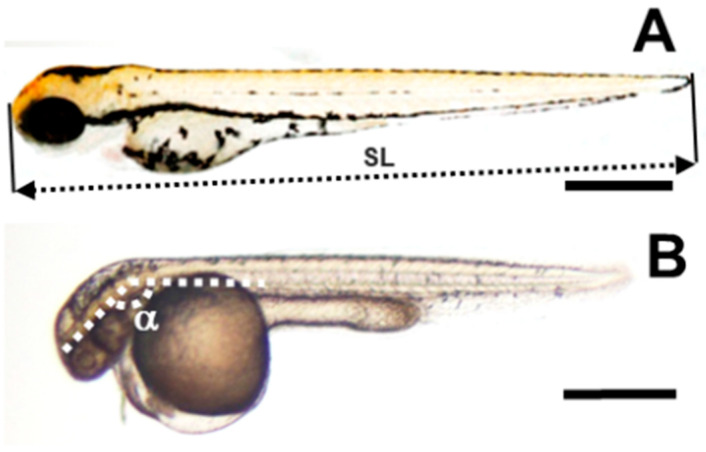
Standard length and head–trunk angle of zebrafish embryos. (**A**) Control untreated zebrafish embryos. The double discontinuous arrow shows the standard length (SL). (**B**) Polysaccharide-treated 72 hpf embryo. α indicates the head–trunk angle as suggested by Kimmel et al. [60]. Bars represent 500 µm.

Several anatomical characteristics were studied to understand how ulvans affect zebrafish embryogenesis. The frequencies of viability, pericardial edema, and hatching were measured daily in zebrafish embryos exposed to increasing ulvan concentrations. As described in the Section 4, other variables, such as the standard length or head–trunk angle (Figure 4), were calculated after digital images were obtained at 72 hpf. Other less frequent anatomical characteristics, such as body abnormalities or short size, body mobility, abnormal head or yolk, curved body or tail, or depigmentation, were eventually annotated when observed.

**Figure 5:**
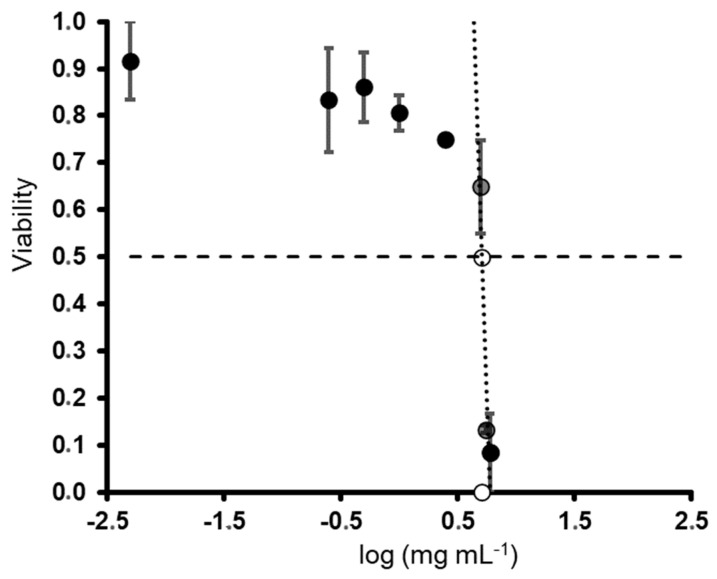
LC_50_ estimation for ulvan polysaccharide treatment of zebrafish 48 hpf embryos. Circles and vertical bars are the means and standard deviations of data from three replicates. Gray, transparent circles are data used to estimate the LC_50_. The empty circle intersects the regression line and 50 % viability. The white circle is the log (LC_50_) estimation. Linear adjustment is y = −7.2237x + 5.6279 (R^2^ = 0.8357).

The increasing ulvan concentrations gradually affected embryo viability. At 48 hpf, the polysaccharides steadily reduced viability (from 100 to 70%) up to about 2.5 mg mL^−1^, at which point a sharp decline in this index was observed (Figure 5). Following the log-linear regression approach [61], we measured an LC_50_ of 5.127 mg mL^−1^.

**Figure 6:**
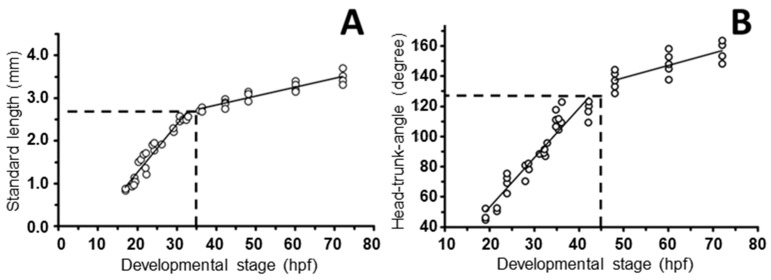
Linear functions used to transform standard lengths and head–trunk angles into hours of post-fertilization development. Graphs show the linear adjustments recovered from approximations to standard length (**A**) and head–trunk angle (**B**) data in Kimmel et al. [60] (Figures 16 and 33, respectively, of Kimmel et al. [60]). Data were obtained using ImageJ 1.50i (nih.gov, accessed on 13 January 2023). The variable transformation functions are (**A**) y = 0.0207x + 2.0153 (R^2^ = 0.8716; *p* ≈ 0.0000) (greater sizes); y = 0.1124x − 0.9848 (R^2^ = 0.931; *p* ≈ 0.0000) (lesser sizes); and (**B**) y = 0.8216x + 97.927 (R^2^ = 0.6126; *p* < 0.00059) (greater angles); y = 3.2744x − 12.452 (R^2^ = 0.9081; *p* < 0.000835) (lesser angles). Discontinuous lines represent the variable transformation limits.

A detailed anatomical description of the 72 hpf embryos exposed to dispersions below the LC_50_ suggests a reduction in the growth rate. To quantify this effect, we measured the standard length and the head–trunk angle, which are two anatomical variables that increase throughout development. The increase in the first variable is gradual over time, whereas the second ranges from 60–70° to 180°. This 180° angle is reached at 72 hpf, and it remains stable through the rest of development [60]. As described in the Section 4, we reproduced data from Kimmel et al. [60] (Figure 6) to obtain four quadratic minimum adjustments that allowed for a linear transformation of these two variables into hours of development (hpf).

**Figure 7:**
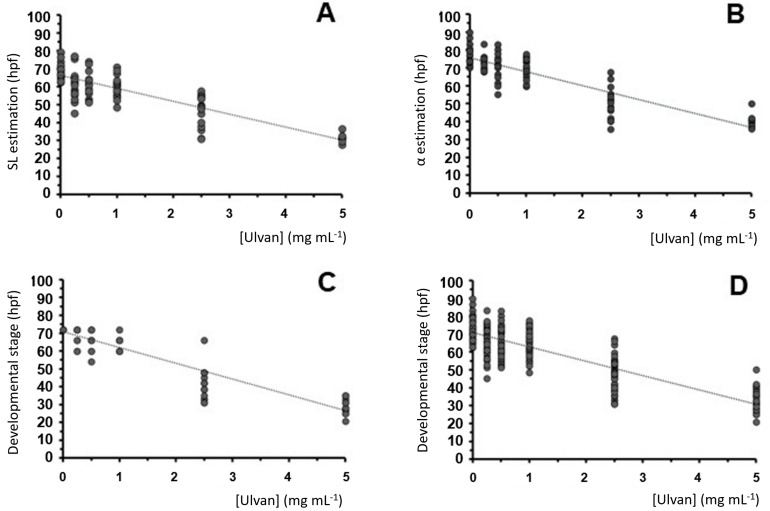
Embryo stage estimation after anatomical variable transformation using data from Kimmel et al. [60] (see Figure 4). (**A**–**C**) Linear regressions of standard length (**A**), head–trunk α angle (**B**), and anatomical-based estimations (**C**) with respect to the ulvan concentration. (**D**) Linear reduction in the compound stage estimation (**A** + **B** + **C**) with respect to the ulvan concentration. The linear adjustments are (**A**) y = −7.2x + 66.234 (R^2^ = 0.7232; *p* ≈ 0.0000), (**B**) y = −7.3294x + 76.026 (R^2^ = 0.8003; *p* < 0.00015), (**C**) y = −8.8x + 70.857 (R^2^ = 0.8738; *p* ≈ 0.0000), and (**D**) y = −7.88x + 71.036 (R^2^ = 0.7479; *p* < 0.000085).

Using this variable transformation method, the stages were estimated from both morphometric variables. The estimated hours post-fertilization showed linear reductions in the concentration of the ulvan polysaccharide dispersions (Figure 7A,B), whereas the embryos exposed to between 0.25 and 1 mg mL^−1^ presented a developmental stage resembling that of the control specimen; the embryos exposed to the highest concentration (5 mg mL^−1^) showed a significant reduction. The linear reduction slopes obtained for both variables were almost identical (7.3 and 7.4 hpf mL mg^−1^) (Figure 7A and Figure 7B, respectively), supporting the initial observation of growth delay. Using this method, the dispersion of the data was high (low R^2^ values) (Figure 7) when compared with the non-transformed variables (see Figure 6A).

Furthermore, a detailed anatomical description of the digital images from each 72 hpf embryo compared to the descriptions provided by Kimmel et al. [60] supported this hypothesis. Several anatomical characteristics (see above) were compared to the data from Kimmel et al. [60] to provide a tentative developmental stage for each embryo. When possible, the vascular pattern was also observed using the *Tg(fli1a:EGFP)y1* transgene to confirm the proposed stage [62]. Almost all the variables supported the occurrence of a growth rate reduction with respect to the ulvan concentration. This anatomy-based estimation also showed a linear decline with regard to the ulvan concentration, having a slightly higher slope of 8.8 hpf mL mg^−1^ (Figure 7C). 

To perform a global stage estimation, we calculated the linear variation based on the summary of our three estimations for each embryo (Figure 7D). This global estimation also showed a linear reduction in ulvan concentration with a slope of 7.8 hpf mL mg^−1^ (Figure 7D). 

The mean value and standard deviation of each stage estimation are also shown in Table 3. All the experimental conditions differ from those of the control, showing an increasing statistical significance with regard to the ulvan concentration (from *p* < 0.05 * to *p* < 0.001 ***) and proportionally higher standard deviations. All these data support the sub-lethal effects of ulvans at concentrations lower than the LC_50_.

In this study, we further dechorionated the embryos and replicated the experiment at a concentration of 2.5 mg mL^−1^ to rule out the potential involvement of hypoxia caused by chorion pore obliteration resulting from ulvan precipitation. In these replicated experiments, no significant modification was found in the mean delay observed (data not shown). 

**Figure 8:**
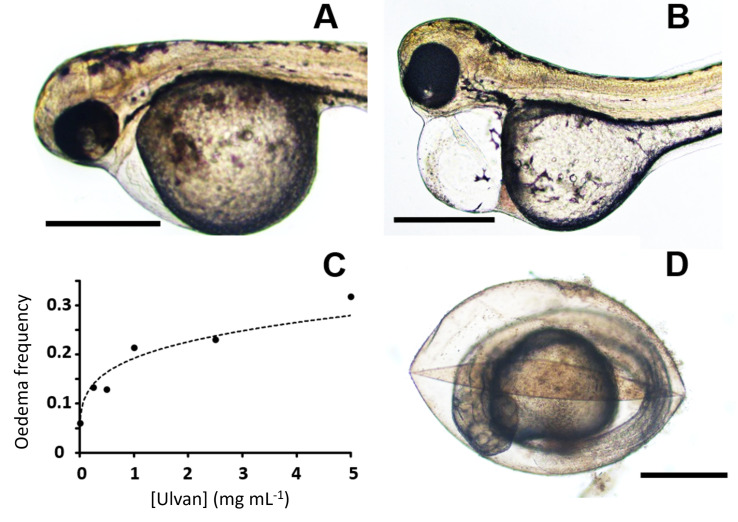
Pericardial edema and chorion lysis increase with the ulvan polysaccharide concentration. Slight (**A**) and significant (**B**) pericardial edemas are seen in zebrafish embryos treated with a 0.25 mg mL^−1^ ulvan concentration. (**C**) Exponential regression of edema frequency versus ulvan polysaccharide concentrations (y = 0.0386x^0.2325^, R^2^ = 0.9351). (**D**) Chorion lysis observed at 2.5 mg mL^−1^ ulvan. Bars represent 500 µm.

Two other toxic effects were frequently observed at sub-lethal concentrations: pericardial edema and chorion lysis (Figure 8). This pericardial edema also augmented in size (Figure 8A,B) along with the increasing ulvan concentration. This effect presented an exponential rather than a linear trend as the ulvan concentration increased (Figure 8C). Larvae with pericardial edemas may also present an apparent increase in yolk sac size. This may be because the yolk is physically displaced by the augmentation of interstitial liquid in the edemas. In our experiments, at concentrations over 2 mg mL^−1^, a slight amount of precipitate could be found over the plastic well and the yolk sac. At these concentrations, a second effect was also observed. As in cell plasmolysis, extra-chorionic hypertonic ulvan solutions generate chorion shrinkage (Figure 8D). This was not observed at lower concentrations. 

## 3. Discussion

Ulvan, the main polysaccharide found on the cell wall of *Ulva* species, is a bioactive compound of great biotechnological interest. Different reviews (see [14,63]) have described the potential of ulvans as an anticancer, immune-modulating, anticoagulant, antiviral, antihyperlipidemic, and antioxidant molecule. Among the bioactivities mentioned above, the anticancer aspect is always highlighted due to the social impact of the disease and, consequently, the need for therapeutic compounds that act selectively on those cells. In 2018, it was estimated that there were almost 290,000, 1,100,000, and 440,000 new cases of melanoma, colon cancer, and leukemia, respectively, and about 61,000, 555,000, and 310,000 deaths worldwide [64]. 

Our study provides further evidence of the anticancer effects of ulvan polysaccharides from *U. rigida*. The anticancer activity of ulvan from different sources is highly variable. Ahmed and Ahmed [21] previously showed that the ulvan from *Ulva lactuca* induced antitumor cytotoxic effects against HepG2 (IC_50_ 55.56 µg mL^−1^) and HCT-116 (IC_50_ 22.65 µg mL^−1^) human cell lines. Thanh et al. [22] also reported a high cytotoxic effect of ulvan polysaccharides on HepG2, MCF7, and HeLa, obtaining IC_50_ values of 29.67 ± 2.87, 25.09 ± 1.36, and 36.33 ± 3.84 µg mL^−1^, respectively. In our study, ulvan exhibited cytotoxic effects against the three human cancer cell lines tested. However, only the HCT-116 cells exhibited sufficient sensitivity to this ulvan in terms of its potential as an anticancer treatment, presenting an IC_50_ of 0.1 mg mL^−1^; however, this was significantly lower than that found by Ahmed and Ahmed [21]. In general, the mechanisms involved in ulvan’s anticancer effect are not fully understood. Some preliminary studies indicate that apoptosis may stimulate programmed cell death or reduce DNA replication and cell proliferation [21,22,65,66]. Other scientists have observed that the polysaccharide’s structure (the number of monosaccharides, glycosidic linkages, sulfate, carboxyl, and hydroxyl groups) might enhance its contact with tumor cells and boost its anticancer effect [67,68,69]. This is supported by the FTIR spectra and monosaccharide composition obtained in our results, in which rhamnose, glucuronic acid, and xylose were the most represented (80.60%, 9.14%, and 4.01%, respectively). Furthermore, the tested molecules must present a specific selectivity for cancer cells over healthy cells to be considered a safe cancer treatment compound. This parameter can be stipulated using the selectivity index (SI). According to Weerapreeyakul et al. [70], a promising, safe compound should present an SI > 3. In our study, ulvan exhibited SI values > 3 on the HCT-116 cells compared to the healthy epithelial cells (40.9 for HACAT and 11.5 for 1064sk). Our work supports the polysaccharide’s potential as a candidate for use in colon cancer treatment. Unfortunately, this promising effect was not observed in the human myeloid leukemia (U-937) or human malignant melanoma (G-361) cells. It is also worth noting that other authors found only extremely low to moderate cytotoxic activity compared to cancer chemotherapy drugs [71,72]. For example, ulvan from *U. intestinalis* demonstrated no cytotoxic effects on sarcoma 180 tumor cells in vitro at 50–800 μg mL^−1^ but reduced the sarcoma 180 tumor weight by 61–71% in mice dosed with 100–400 mg kg^−1^ [73]. Furthermore, immunological organs (such as the thymus and spleen) were increased in ulvan-treated mice, suggesting that the polysaccharide’s anticancer activity stems from its immunomodulatory function. In summary, the anticancer activity of ulvans appears to be mediated through one or more routes, which include enhancing cancer cell apoptosis, decreasing cancer cell growth, and activating the innate immune response. In addition, the affected pathways are altered depending on the ulvan source and/or structure. More research is needed to investigate the structural and chemical components that influence ulvan’s ability to reduce the number of cancer cells and to figure out the relationship between them.

To further study the toxicity effects of ulvans in vivo, we conducted a zebrafish embryo toxicity test (ZFET). In general, our data agree with the idea of using zebrafish embryos as an effective system to evaluate the effects of glycans in relatively short procedures. Previous studies [74] suggest a close homology between zebrafish embryogenesis and human carcinogenesis, thus supporting its use as a screening assay to evaluate potential anticancer compounds. In this sense, Rusdi et al. [74] and other studies [25,56] have already evaluated the toxicity of algal and fungal polysaccharides on zebrafish embryos, suggesting either absent [74] or low [25,56,74] toxicity. Nevertheless, the measurement of the LC_50_ of these polysaccharides has revealed a variable susceptibility of early zebrafish embryos to their presence in the embryo medium.

Although fucoidan from *Fucus vesiculosus* showed no LC_50_, alginate LC_50_ was measured at 245 µg mL^−1^ after 24 h [74]. A decline in zebrafish embryo survival (from about 75% at 48 hpf to 23% at 72 hpf) was also reported when using 5 mg mL^−1^ of exopolysaccharides from a fungus species, *Ganoderma applanatum*. In contrast, its endopolysaccharides showed a viability decline from about 75% at 72 hpf to 32% at 96 hpf [56]. These results suggest an LC_50_ higher than that found for *F. vesiculosus* alginate at 24 h or ulvans in our study at 48 hpf (5.2 mg mL^−1^). Moreover, 5 mg mL^−1^ of natural mycelial biomass from *Lignosus rhinoceros* showed a survival rate decline between 72 hpf (65%) and 96 hpf (5%), while 5 mg mL^−1^ of exopolysaccharides showed a 50 % zebrafish mortality rate at 72 hpf [25]. In these cases, fungal exopolysaccharides presented slightly higher LC_50_ values than those found for ulvans, whereas those of endopolysaccharides and mycelial extracts were even higher. These effects mimic the anticancer activity discussed above and further support the case for ulvans as a candidate cancer treatment compound. From this comparison, the ulvans tested in our study seem to be a better option for a potential anticancer product than others from brown algae [74] or fungi [25,56] species, although they are less effective than *Fucus* alginate [74].

A teratogenic effect on zebrafish pigmentation has also been described at lower polysaccharide concentrations, molecularly related to the interference of several tyrosine kinase downstream effectors in carcinogenesis [74,75]. In our study, ulvans between 0.1 and 5 mg mL^−1^ linearly delayed pigmentation and embryonic development at a rate of 7.6 hpf per mg mL^−1^. Partial hypoxia generated by the obliteration of chorionic pores [76] from ulvan precipitates was initially considered the potential cause of this delay. Our results ruled out this hypothesis because of the delay observed in our experiment on dechorionated embryos.

Recent studies have proposed alternative assays to the ZFET test, such as the zebrafish embryo acute toxicity test (ZET) or the General and Behavioral Embryo Toxicity Assay [28]. In both tests, several phenotypes have been proposed to describe toxicity. The most critical phenotypes in these tests are developmental abnormalities; a short body size; body mobility and position; a slow heartbeat; pericardial, yolk or head edemas; an abnormally sized or darkened head, yolk or liver; a curved body or tail; or pigment abnormalities [28]. Our study associated a short body size, an abnormal head size, and pigment abnormalities with signs of developmental delay, all of which are phenotypes included in the scoring panel of the ZET [28] and ZFET [25] tests. Three different estimations of this effect were evaluated to support our hypothesis: anatomical descriptions, the standard length, and the head–trunk angle. This association is not explicitly included in the ZET and ZFET assays. Parallel to our study, we conducted experiments with five other polysaccharides from algal and fungal species in search of an appropriate positive control [77]. In certain instances, this developmental delay effect has also been observed, suggesting a new toxic phenotype produced by increasing algal and fungal polysaccharide concentrations (Abdala-Díaz and Marí-Beffa, in preparation). Furthermore, the variables used to support the occurrence of this phenomenon always show high dispersion estimates. This suggests a potential variable susceptibility of zebrafish embryos to this type of substance in the culture medium. This new growth delay effect of algal polysaccharides may also be tested over cancer and zebrafish development in xenograft experiments [78]. These experiments are currently underway and will be published elsewhere. In our results, no other teratogenic defects were consistently associated with the ulvan concentration.

Finally, new osmolar and toxicity effects were observed at the highest viable concentrations, such as chorion lysis and pericardial edema. In principle, chorion lysis may be caused by osmolar imbalance and increased water loss. Moreover, in various substances, pericardial edema is a well-established toxicity symptom seen in zebrafish embryos [79]. In our study, we did not test whether zebrafish toxicity is associated with inflammation or any other cell stress process. In principle, the food, feed, fertilizing, and biomedical modulatory effects of ulvan [12,14] could be accompanied by relevant metabolic perturbations that might induce toxicity and affect osmolar imbalance and embryonic growth as the concentration increases from 0.25–1 mg mL^−1^. 

The lowest ulvan concentration in our study, 0.25 mg mL^−1^, is higher than the 0.05 mg mL^−1^ of *Spirulina maxima* pectins, a concentration within the range of the above-mentioned cytotoxic IC_50_ indexes [21,22], which stimulates larval fin regeneration [58]. Studies on the immunomodulatory effects of *U. rigida* ulvans in model organisms are also in progress to support the beneficial effects of these compounds at concentrations within the colon cancer cytotoxic LC_50_ range (data not shown). 

## 4. Materials and Methods

### 4.1. Ulvan Preparation

*Ulva rigida* was cultivated in 500 L aerated semi-circular fiberglass tanks under natural outdoor conditions at the facilities of the Andalusian Institute of Blue Biotechnology and Development (IBYDA) at Malaga University (Málaga, Spain). The algal biomass was harvested weekly, washed gently with abundant natural seawater, and dried at 60 °C for 24 h. Afterwards, the biomass was milled to obtain a fine powder (50 µm) and stored at −20 °C. Ulvan was then extracted from the powdered sample using the ethanol precipitation method according to Béress et al. [80]. The *U. rigida* powder was submerged in 95% ethanol (a biomass-to-ethanol ratio of 1:10) until de-pigmentation was apparent. Subsequently, the de-pigmented biomass was suspended in distilled water and heated at 90 °C for 2 h. The solution was then centrifuged for 15 min at 6000 rpm at room temperature. The supernatant was concentrated to 1/5th of the original volume. Following this, five times the volume of 95% ice-cold ethanol was added to the concentrated solution and stored at 4 °C. The precipitate that formed was collected via centrifugation at 12,000 rpm for 10 min at 4 °C, washed twice with absolute ethanol, and freeze-dried.

### 4.2. Chemical Composition and Structure of Ulvans

#### 4.2.1. Fourier-Transform Infrared Spectroscopy (FTIR)

The FTIR spectra of the *U. rigida* ulvans were obtained by pressing 13 mm diameter self-supporting pressed discs comprising a mixture of ulvans and KBr (1% *w*/*w*) with a hydraulic press at a force of 15.0 tcm^−2^ for 2 min. The FTIR spectra were obtained in the 400–4000 cm^−1^ region using a Thermo Nicolet Avatar 360 IR spectrophotometer (Thermo Electron Inc., Franklin, MA, USA), having a resolution of 4 cm^−1^, with a deuterated triglycine sulfate (DTGS) detector and OmnicTM 7.2 software (bandwidth of 50 cm^−1^ and an enhancement factor of 2.6). Thermo Nicolet OMNIC software was used for baseline correction to smooth the baseline of each spectrum. To compare the sample spectra to those in the spectral collection, the OMNIC correlation algorithm was used.

#### 4.2.2. Gas Chromatography–Mass Spectrometry (GC–MS)

The GC–MS of the ulvans was determined following the methodology described in detail by Parra-Riofrío et al. [81,82]. In brief, GC–MS analyses were carried out using a Trace GC gas chromatograph (Thermo Fisher Scientific, Franklin, MA, USA), a Triplus RSH autosampler (Thermo Fisher Scientific, Franklin, MA, USA), and a DSQ quadrupole mass spectrometer (Thermo Fisher Scientific, Franklin, MA, USA). The identification of monosaccharides in the polysaccharide samples was carried out by comparing the retention time and mass spectra of monosaccharide standards, previously analyzed under identical conditions (glucose, galactose, mannose, arabinose, xylose, rhamnose, ribose, fucose, galacturonic acid, and glucuronic acid). The compounds were identified by comparing the mass spectra with those in the National Institute of Standards and Technology (NIST 2014) library.

### 4.3. Cytotoxic Effect Assay

Five human cell lines, 1064sk (human fibroblasts; CIC cell bank of CIC of the Universidad de Granada, Spain), HACAT (immortalized human keratinocytes; ATCC, Manassas, VA, USA), U-937 (human leukemia cell line; ATCC, Manassas, VA, USA), G-361 (human malignant melanoma; ATCC, Manassas, VA, USA), and HCT-116 (colon cancer cell line; ATCC, Manassas, VA, USA), were used for the assays. The cells were cultured in either Dulbecco’s Modified Eagle’s Medium (DMEM) (Capricorn Scientific, Ebsdorfergrund, Germany, ref. DMEM-HPSTA), for the 1064sk, U-937, and HCT-116 cells, or in RPMI-1640 medium (BioWhittaker, ref. BE12-167F), for the HACAT and G-361 cells, both containing 10% Fetal Bovine Serum (FBS, Biowest, ref. S1810-500), 1% penicillin–streptomycin solution 100× (Capricorn Scientific, ref. PS-B), and 0.5% amphotericin B (Biowest ref. L0009-100). The cells were maintained sub-confluent at 37 °C in humidified air containing 5% CO_2_.

The cytotoxic effect on the cell lines mentioned above was measured using an MTT assay. The cells were incubated independently in 96-well plates, containing 1 × 10^4^ cell/well for 1064sk and 6 × 10^3^ cell/well for the other cell lines, with different ulvan concentrations (ranging from 0.009 to 5 mg mL^−1^) at 37 °C in a humid atmosphere with 5% CO_2_ for 72 h. As a control, the same cell lines were used without treatment. The trial was carried out following the method proposed by Abdala-Díaz et al. [83]. The cytotoxicity was calculated, and it is expressed as the inhibition concentration at 50% (IC_50_ values). The analyses were carried out in three independent experiments. In addition, the selectivity index (SI) was calculated as the ratio of dividing the IC_50_ value of the healthy cell lines by that of the cancer cell lines.

### 4.4. Zebrafish Husbandry and Embryo Collection

The zebrafish (*Danio rerio*) embryos were the offspring of mating AB wild-type and/or *Tg(fli-1: EGFP) y1* adults. The adults were obtained from the breeding stock at the fish facilities belonging to the Centre of Experimentation and Animal Behavior at the University of Málaga, where they were cultured in a 12:12 h light:dark photoperiod following standard procedures [61,62]. The adults were the offspring of fish obtained from the Zebrafish International Resource Centre (ZIRC, Eugene, OR). The eggs were collected after fertilization and then bleached, washed, and incubated at 28 ± 0.1 °C in a Petri dish with an embryo medium. The fish were handled in accordance with notification A/ES/12/I-22 (activity A/ES/12/24) of the National Laws. The Universidad de Málaga Bioethics Commission approved the experiments as part of the grants BIO2014-56092-R and UMA18-FEDERJA-274. 

### 4.5. Zebrafish Embryo Toxicity Assay

The AB wild-type or *Tg(fli-1:EGFP)y1* embryos were placed in 96-well plates (1 fish per well using 300 µL embryo medium) at 4 h post-fertilization (4 hpf) [84]. Each replicate comprised 8 embryos immersed in the embryo medium with a specific polysaccharide concentration (from 0.25 to 6.5 mg mL^−1^), comprising 56 embryos per experiment and including both negative and positive controls. The embryos were immersed for three days following the ZFET procedure [25] without further modifications. The concentrations were obtained by diluting 5 or 10 mg mL^−1^ stock solutions. The stock solutions were prepared from polysaccharide lyophilized powder and stored at 4 °C after preparation. The embryo medium served as the negative control [84]. Polysaccharides from the macroalga *Sarcopeltis skottsbergii* and the fungus *Calvatia* (in preparation) were used as positive controls in parallel experiments. Both polysaccharides presented a relevant LC_50_ and a significant induction of growth delay at lower concentrations. LPS was discarded as a positive control due to its well-known inflammatory effects [77]. Each experiment was run at least three times with a minimum of 24 embryos for each experimental condition. Basic statistics were obtained from the data described below. After the experiments, the live embryos were euthanized using MS-222 (0.2 mg mL^−1^) over-anesthetization and stored as organic waste following the University of Málaga procedures. 

### 4.6. Phenotypic Analysis

In the ZFET, several phenotypes were annotated to support the toxic effects of the reagents tested. The following were used in this test: embryo viability, hatching, and heart rate [25]; tail malformations; or the absence of fins, the gut, or melanophores [56]. Other phenotypes, such as the standard length, head–trunk angle [60], or pericardial edema, were estimated for each living specimen at the end of the experiment. Additional characteristics were used for an anatomical comparison with a standard embryological atlas [60]; these were the shape of the eyes, cochlea, and pec and tail fins; the form and size of the yolk sac, the notochord, and the craniofacial skeleton; and the pigment distribution (see Table 3 for the number of measured embryos). The analysis was performed under a magnifying microscope (Nikon SMZ-445 model) or using digital images obtained with a Nikon Microphot-FX Fluorescence Research Microscope with a Nikon DS-L1 digital camera. The lethal concentration that kills 50% of the sample (LC_50_) was estimated from mortality/viability data following a linear regression test [61]. To estimate the growth delay, the actual age of the embryos was compared to three different stage estimations made from embryo anatomy, the standard length, and the head–trunk angle. The last two variables were transformed into a potential developmental stage using data from Kimmel et al. [60] (see the Results Section 2.2). The quantitative variables were measured from digital images using the ImageJ 1.50i settings (National Institutes of Health, Bethesda, MD, USA) and verified in a single-blind evaluation.

### 4.7. Statistical Analysis

Statistical differences between the means were calculated using the Student *t*-test. Linear regressions were calculated using a least squares analysis. The statistical analyses were performed using the Statistical Package for the Social Sciences software (IBM, SPSS-version 25), Statgraphics software (Statgraphics Technologies, Inc., The Plains, VA, USA), or the Excel program (Microsoft Office, Windows 11, Redmond, WA, USA). Significant differences were noted when *p* < 0.05 (*), *p* < 0.01 (**), and *p* < 0.001 (***). 

## 5. Conclusions

Our in vitro study supports the potential use of polysaccharides extracted from *U. rigida* as candidates for treating human colon cancer. This is further supported by our zebrafish toxicity and phenotypic assay. These in vivo tests revealed the side effects of these molecules on zebrafish development, such as developmental abnormalities and delay. Perturbations of cancer or development-associated signaling pathways may be underlying these phenocopies as previously suggested by other authors. Ulvans may be a promising, safe compound at concentrations below 0.1–0.2 mg mL^−1^.

## Figures and Tables

**Table 1:** Percentage of monosaccharides for ulvans extracted from *U. rigida*.

Monosaccharide	%
Rhamnose	80.60
Glucuronic acid	9.14
Xylose	4.01
Glucose	3.78
Galactose	2.48

**Table 2:** Selectivity index (SI) of *U. rigida* polysaccharides.

	Selectivity Index
HACAT/HCT-116	40.9
1064sk/HCT-116	11.5
HACAT/U-937	1.8
1064sk/U-937	0.5
HACAT/G-361	0.9
1064sk/G-361	0.3

**Table 3:** Embryo stage estimation following the anatomical features.

Ulvan (mg mL^−1^)	Head–Trunk Angle (hpf)	Standard Length (hpf)	Developmental Stage (hpf)	Mean Estimation (hpf)
5	40.25 ± 3.55 ***	31.56 ± 2.15 ***	29.90 ± 4.23 ***	33.91 ± 5.56 (13) ***
2.5	49.73 ± 8.94 ***	47.03 ± 8.50 ***	41.64 ± 8.46 ***	46.13 ± 4.12 (16) ***
1	70.10 ± 5.34 ***	59.98 ± 5.56 ***	63.14 ± 3.61 ***	64.41 ± 5.18 (19) ***
0.5	70.62 ± 7.78 **	61.24 ± 6.92 ***	66.80 ± 5.49 **	66.22 ± 4.72 (15) **
0.25	73.06 ± 3.86 **	61.70 ± 8.91 **	69.88 ± 4.21 *	68.21 ± 5.86 (16) **
0	77.61 ± 5.28	69.60 ± 5.02	72.00 ± 0.00	73.07 ± 4.11 (18)

The results are expressed as the mean and standard deviation. *p* < 0.05 (*); *p* < 0.01 (**); *p* < 0.001 (***). Underlined symbols represent non-parametric analysis. N = 97, 99, and 107 for the head–trunk angle, SL, and developmental stage estimations, respectively. The total number of embryos used to calculate the mean estimation are placed in parentheses (total: 97).

## Data Availability

The data are contained within the article.
